# MRI features of ERSA (exercise-related signal abnormality) lesions in professional soccer players

**DOI:** 10.1007/s00256-021-03857-x

**Published:** 2021-07-06

**Authors:** James S. B. Kho, Rajesh Botchu, Alison Rushton, Steven L. James

**Affiliations:** 1 Imaging Department, Royal Orthopaedic Hospital, Birmingham, B31 2AP UK; 2 Imaging Department, Bristol Royal Infirmary, Bristol, BS7 0QA UK; 3 School of Sport, Exercise and Rehabilitation Sciences, University of Birmingham, Birmingham, UK; 4 School of Physical Therapy, Western University, Elborn College, Ontario, N6G 1H1 Canada

**Keywords:** Muscle injury, Magnetic Resonance Imaging, Athletic injury, Thigh, Soccer

## Abstract

**Objective:**

This study aims to describe the prevalence, anatomy and morphology of ERSA (exercise-related signal abnormality) lesions, a previously undescribed pattern of muscle signal changes on MRI in professional soccer players with suspected acute thigh muscle injury.

**Methods:**

A multicenter retrospective review was performed of 287 MRIs of professional soccer players referred for suspected acute thigh injury from August 2017 to February 2020. MR images were reviewed for muscle signal abnormalities corresponding to a peritendinous ovoid region or a subfascial ring of faint increased signal on fluid-sensitive MR images. Imaging features including anatomical site, morphology, and craniocaudal length were recorded. Concomitant acute muscle injury was graded in accordance with the British Athletics Muscle Injury Classification (BAMIC).

**Results:**

ERSA lesions comprising a peritendinous ovoid region, a subfascial ring, or both, were identified in 40 muscles across 31/287 studies (10.8%). These lesions had a mean length of 15.8 cm and were predominantly located in the proximal or mid-portions of muscles. Affected muscles were rectus femoris (*n *= 22), adductor longus (*n *= 11), semitendinosus (*n *= 6) and biceps femoris (*n *= 1). 21/31 studies (67.7%) had a BAMIC grade 1–4 injury in a separate muscle, which were largely (81%) in a separate anatomic compartment or contralateral.

**Conclusion:**

ERSA lesions were evident on MRI in 10.8% of our cohort of professional soccer players referred for suspected acute thigh muscle injury. Characteristic morphology and the longitudinal length (mean 15.8 cm) distinguish ERSA lesions from recognized patterns of acute muscle injury.

## Introduction

Muscle injuries are a common occurrence in sports at professional and amateur levels, although frequencies of muscle injury vary between sports [1–5]. At the professional level, muscle injuries account for between 15 and 40% of all sports injuries in international championships, and most commonly involve the lower limb [4, 5]. Muscle injuries are an important cause of time lost to both training and play, accounting for a quarter of injury related absences in professional soccer [6]. There has been recent interest in the grading and classification of muscle injuries to guide treatment and return to play [7–9]. Different classifications of muscle injury exist including the Munich Classification, Chan and the British Athletics Muscle Injury Classification (BAMIC) [9–11]. The BAMIC classification system, in particular, has gained widespread use in professional sport over the past few years. It describes the MRI features of different grades (0–4) of muscle injury, and sub-classifies these grades according to the site of injury (myofascial, musculotendinous or intratendinous) [11].

The senior author had recognized a distinct pattern of muscle signal changes on MRI that can be observed in professional soccer that does not conform to the typical patterns of muscle injury in the literature or those described within the BAMIC classification system. These muscle signal changes specifically are of a peritendinous ovoid region or a subfascial ring of faint increased signal on fluid-sensitive MR images. These imaging features are distinct on MRI to the typical feathery pattern of edema that characterizes an acute muscle strain injury. However, to date, there is no description of these muscle signal changes to enable their distinction and to potentially inform decision-making regarding injury management and rehabilitation. This study aims to describe the MRI appearances of these muscle signal changes in a cohort of professional soccer players who were imaged with a suspected acute thigh muscle injury, and to investigate the frequency and anatomical locations of these findings.

## Methods

### Design

A retrospective case review of consecutive referrals for MR imaging from an anonymized database was performed. The database consists of MR images of professional soccer players performed at several private clinics. These cases were identified through searching a Horos Project imaging database.

### Study cohort

A search was performed through the database for consecutive MRI studies of professional soccer players referred for suspected acute thigh muscle injury over a 30-month period from August 1, 2017 to February 1, 2020. Specific inclusion criteria to the study cohort were age of 16 years or older, referral for suspected thigh muscle injury, and MRI within 48 h of the index injury. Exclusion criteria were incomplete MR imaging defined as the absence of fluid-sensitive MRI sequences in any of the 3 orthogonal planes. A suspected acute muscle injury was defined for the purposes of this study as persistent pain when clinically reassessed within 24 h of the injury that occurred either in training or in an in-game incident. Demographic information including the age, gender and side of injury was collected for each MRI study.

### Ethical considerations

Ethical approval was obtained from the University of Birmingham Ethics Committee (Ref: ERN_19-1936). The MRI examinations had already been performed for the assessment of muscle injury at a number of institutions in the UK. A formal radiological report had already been provided to the athlete and referrer. Ethical approval was granted for the retrospective review of MR images included in this study. MR images were stored in anonymized Digital Imaging and Communications in Medicine (DICOM) format. The DICOM format stores participant information in a separate header from imaging data. Information in this header was anonymized (names and identifying information removed) in DICOM files available to investigators. Data were stored on an encrypted computer for analyses. Data will be stored for 10 years in accordance with the University’s Research Governance procedures, after which the data for this study will be disposed of securely by the senior author.

### Image analysis

Each MRI study was independently reviewed by 2 musculoskeletal radiologists RB and SLJ, each with over 10 years of experience in sports injury imaging. The MRI studies were performed at a number of different institutions but all MRI protocols incorporated fluid sensitive (short TI inversion recovery or fat-suppressed intermediate-weighted proton-density) sequences in 3 orthogonal planes enabling a description in both the axial and longitudinal plane. MRI protocols also typically included axial T1 or intermediate-weighted PD sequences.

**Fig. 1:**
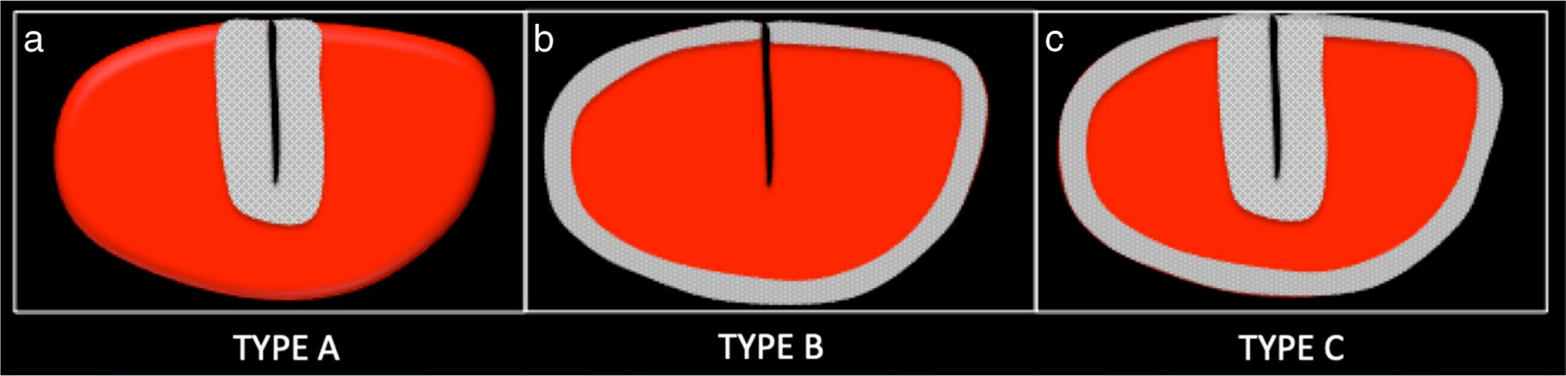
Schematic diagram illustrating the 3 types of ERSA lesions encountered. The grey shading indicates the location of increased signal intensity on fluid sensitive images. **a**. Type A lesion with peritendinous ovoid region of increased signal. **b**. Type B lesion with subfascial ring of increased signal. **c**. Type C lesion with peritendinous ovoid region and subfascial ring of increased signal

The images from each MRI study were reviewed for muscle signal abnormalities on MRI that correspond to a peritendinous ovoid region or subfascial ring of faintly increased signal on fluid-sensitive images, as determined a priori. No previous terminology exists in the literature for these signal abnormalities so the authors’ proposed term — exercise-related signal abnormality (ERSA) — is used for these signal abnormalities to facilitate presentation of the methods and results. Where ERSA lesions are present, the side, anatomical site, morphology, craniocaudal length, location within the muscle (proximal, middle or distal thirds) and number of muscles involved were recorded. ERSA lesions were then sub-classified according to the anatomic appearances of the region of increased signal when viewed on axial fluid sensitive images, namely: type A for peritendinous ovoid region, type B for the subfascial ring, and type C where both co-exist (Fig. 1). The site of any concomitant acute muscle injury was recorded for each study, and graded in accordance with the BAMIC classification (Table 1) [11]. Any discrepancies between reviewers were resolved by consensus review.

**Table 1:** Summary of BAMIC classification system

BAMIC grade	Imaging features		
	Craniocaudal length of high signal change	Percentage muscle cross-sectional area of high signal change	Fibre disruption
Grade 0	Not applicable	Not applicable	Not applicable
Grade 1	< 5 cm	< 10%	< 1 cm
Grade 2	5 cm to 15 cm	10–50%	1 cm–5 cm
Grade 3	> 15 cm	> 50%	> 5 cm
Grade 4	Any	Any	Complete discontinuity of muscle or tendon

Grade 0 injuries demonstrate either normal MRI appearances or patchy signal change. Where there are imaging features of different grades, the assigned grade is the highest grade for which imaging features are present. A grade suffix (a–c) is assigned based on myofascial, myotendinous or intratendinous location

For MRI studies that demonstrated ERSA lesions, a subgroup analysis to determine the chronicity of ERSA lesions was performed. The imaging database was searched for any prior or subsequent MRIs of the thighs within one year of the MRI demonstrating ERSA. Where prior or subsequent MRIs were available, the presence or absence of ERSA in the prior or subsequent MRI, and time interval between MRIs were recorded.

### Data analysis

Anonymized data on MRI findings and demographics was collated in Microsoft Excel and statistical analysis was performed with SPSS Statistics 25. Descriptive analysis of data included mean (SD) where data reflected a normal distribution, as confirmed with the Shapiro–Wilk test. Inferential statistics using the chi-squared test enabled exploration of the relationships among patterns of signal abnormality. A p-value of 0.05 was taken to be the level of statistical significance.

## Results

### Patient characteristics

A total of 287 MRI studies referred for suspected acute thigh muscle injury which met the inclusion criteria were reviewed. No studies met exclusion criteria. Out of these 287 studies, 31 studies (10.8%) demonstrated at least one ERSA lesion. Athletes in these 31 studies had a mean age of 24.3 years (SD = 5.8 years), and all but one were male. There were 18 right-sided studies and 13 left-sided, in one case the lesions were bilateral. 10 cases were identified on 3 T MRI studies with the remainder being performed at 1.5 T.

### MRI features and distribution of ERSA lesions

In 22 studies, ERSA lesions were present in a single muscle, and in the remaining 9 studies, these signal abnormalities were present in 2 muscles. In total, therefore, ERSA lesions were present in 40 muscles. The most commonly affected muscle was rectus femoris (*n* = 22) followed by adductor longus (*n* = 11), semitendinosus (*n* = 6) and the long head of biceps femoris (*n *= 1).

**Fig. 2:**
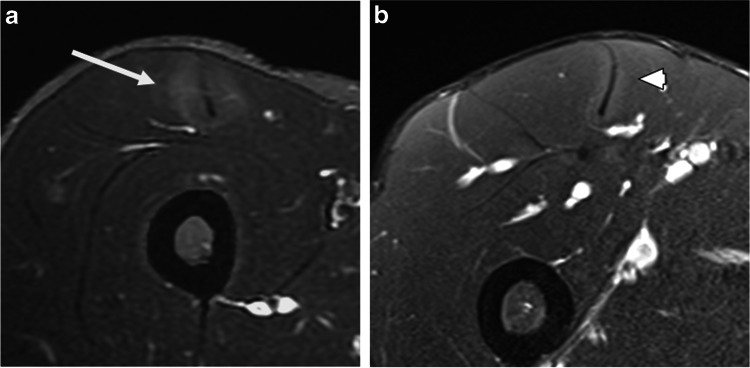
Type A ERSA lesion. **a**. Axial PDFS image of the thigh demonstrating peritendinous ovoid region of increased signal around the central tendon of rectus femoris (white arrow). **b**. Axial PDFS image of the thigh in a different patient demonstrating similar findings of peritendinous increased signal (white arrowhead) in rectus femoris muscle

**Fig. 3:**
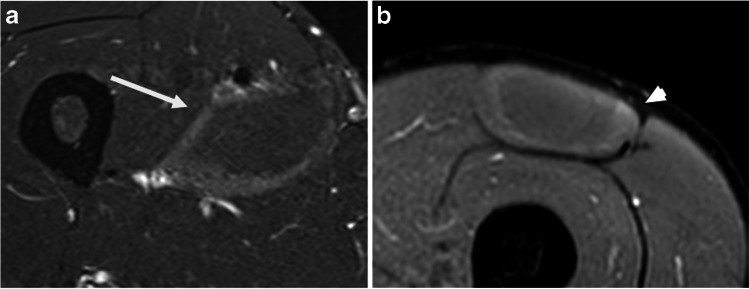
Type B ERSA lesion. **a**. Axial PDFS image of the thigh demonstrating a subfascial ring of increased signal in adductor longus (white arrow). **b**. Axial PDFS image of the thigh in a different patient illustrating a subfascial ring of increased signal in rectus femoris muscle (white arrowhead)

**Fig. 4:**
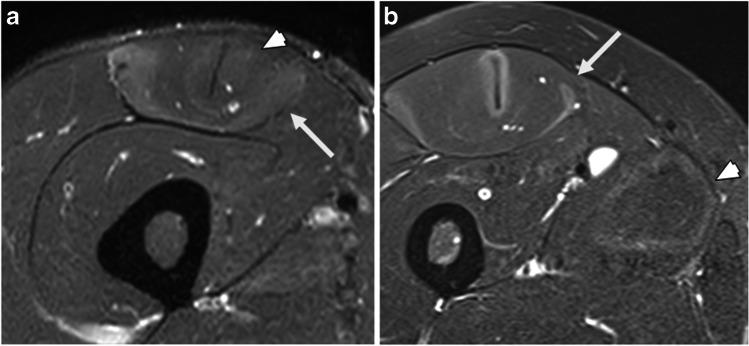
Type C ERSA lesion. **a**. Axial PDFS image of the thigh illustrating a peritendinous ovoid region (white arrowhead) and subfascial ring (white arrow) of increased signal in rectus femoris. **b**. Axial PDFS image of the thigh in a different patient demonstrating a type C lesion in rectus femoris (white arrow) and a type B lesion in adductor longus (white arrowhead)

Among these 31 studies, a peritendinous ovoid region of increased signal was seen in at least one muscle (ERSA Type A) in 21 studies (67.7%), and the subfascial ring of increased signal (ERSA Type B) in 23 studies (74.1%). (Figs. 2 and 3) There was a statistically significant association between these two patterns of signal abnormality (χ2 = 89, df 1, *n *= 287, *p *< 0.0001), and in 9 studies, these two signal abnormalities were present in the same muscle (ERSA Type C) (Fig. 4). The distribution of these ERSA, subclassified as type A-C, is depicted in Table 2.

**Table 2:** Distribution of ERSA lesions, by muscle and subtype

Muscle affected	Number of ERSA lesions		
	Type A	Type B	Type C
Rectus femoris	10	3	9
Adductor longus	-	10	1
Semitendinosus	-	6	-
Biceps femoris	1	-	-

*ERSA*, exercise-related signal abnormality

**Fig. 5:**
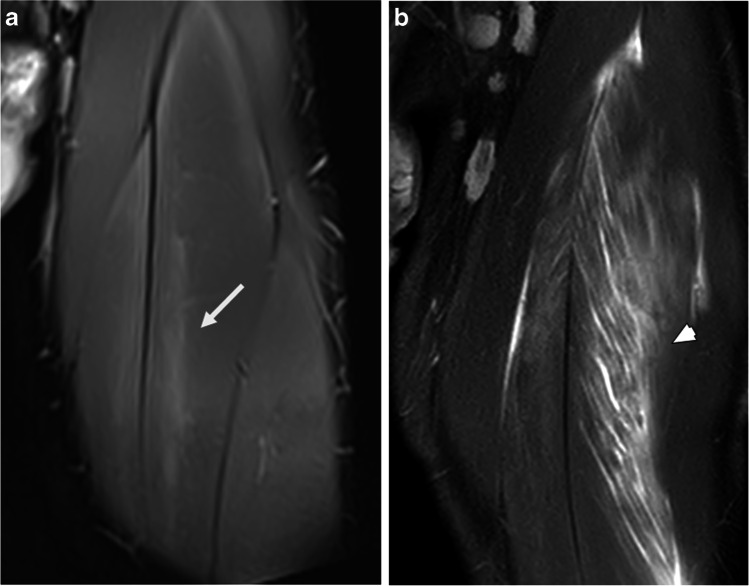
Craniocaudal extent of ERSA and comparison with acute muscle injury. **a**. Coronal PDFS image of the thigh showing the craniocaudal extent of a type A ERSA lesion in rectus femoris (arrow). **b**. Coronal PDFS image of the thigh in a different patient illustrating an acute muscle injury of rectus femoris with similar craniocaudal length but with typical ‘feathery’ edema of acute muscle injury (arrowhead)

The craniocaudal length of ERSA ranged from 6 to 27 cm, and demonstrated a normal distribution (W(40) = 0.965, *p* = 0.253) with a mean length of 15.8 cm (SD = 5.1 cm) (Fig. 5). These predominantly involved the proximal or mid portions of muscles. Of 40 ERSA lesions, 14 were proximal, 18 were mid-portion and 7 extended from the proximal to mid-portion; only 1 was located distally within a muscle.

### Frequency of concurrent BAMIC grade 1–4 acute muscle injury

Twenty-one studies (67.7%) had a concurrent BAMIC grade 1–4 injury in a thigh muscle, most frequently of biceps femoris or rectus femoris. There were 27 ERSA lesions in these 21 studies. 3 ERSA lesions (11.1%) were contralateral to the site of the muscle injury. 19 ERSA lesions (70.3%) were ipsilateral to, but in a separate muscle compartment to the BAMIC grade 1–4 injury, and 5 (18.6%) were in the same compartment (Table 3).

**Table 3:** Concomitant ERSA and BAMIC injury: Anatomic compartment of ERSA lesions relative to the anatomic compartment of BAMIC grade 1–4 injury, when ipsilateral (*n* = 24)

Compartment of ERSA lesion	Compartment of ipsilateral BAMIC grade 1–4 injury		
	Hamstring	Quadriceps	Adductor
Hamstring	4	0	0
Quadriceps	10	1	2
Adductor	4	3	0

3 further ERSA lesions were contralateral to BAMIC grade 1–4 injury

ERSA, exercise-related signal abnormality; BAMIC, British Athletics Muscle Injury Classification

### Temporal changes of ERSA lesions

Of the 31 studies demonstrating ERSA, 8 had a further MRI of the thigh within one year. In 6 cases, there was a subsequent MRI study ranging from 19 days to 7 months later. In the other two cases, there was a prior MRI study ranging from 7 to 9 months earlier. All 8/8 cases did not demonstrate an ERSA lesion in the prior or subsequent MRI, implicating that ERSA is not a chronic lesion.

## Discussion

### Exercise-related signal abnormality as an imaging entity

A significant proportion (10.8%) of MRI studies performed for suspected muscle injury in our cohort exhibited either a peritendinous ovoid region and/or a subfascial ring of faint increased signal on fluid-sensitive images in at least one muscle. Despite the relative frequency of these findings, these appearances have never previously been recognized as a distinct imaging pattern or entity in the literature, to the authors’ knowledge. To facilitate recognition and discussion of this entity, the authors propose the terminology exercise-related signal abnormality (ERSA lesion) for these MRI signal changes.

The terminology ERSA lesion is proposed for the presence of either the peritendinous ovoid ring alone (type A), the subfascial ring alone (type B), or both together (type C). Given the statistical association between the peritendinous ovoid ring and subfascial ring of altered signal change, and the co-occurrence in the same muscle in 9 instances in our cohort, it appears reasonable to consider both findings as different MRI manifestations of a single imaging entity.

### Distinction of ERSA lesion from acute muscle injury

Muscle injuries can be divided into injuries resulting from direct trauma applied to the muscle, and indirect injuries resulting from excessive tension during muscle contraction, particularly during eccentric contraction [9]. Direct injuries such as muscle contusions and lacerations will not be discussed further as the clinical context and imaging appearances are different, and should not pose diagnostic confusion from ERSA lesions. Indirect muscle injuries comprise muscle strains and tears that demonstrate a continuum of appearances depending on the extent of fiber and tendon involvement.

ERSA lesions are characterized by a faint increase in signal intensity on fluid-sensitive images that is less pronounced than indirect muscle injuries and lacks the feathery edema pattern that is typically seen with an acute muscle strain. (Fig. 5) Unlike muscle tears, there is no focal disruption in muscle fibers, nor architectural distortion of the muscles. Furthermore, there is no tendon involvement. Despite the relatively subtle findings on axial imaging, ERSA lesions demonstrate a prolonged cranial-caudal extension, with a mean craniocaudal length of 15.8 cm in this study. Lastly, rapid resolution of ERSA lesions, within 19 days after the initial MRI in one case, would be unusual for an acute muscle injury where the abnormality was seen over such a long longitudinal length.

It is critical to appreciate the difference in imaging appearances of ERSA lesions versus an acute muscle strain to avoid overestimating the significance of the imaging abnormality, particularly if classifying according to the BAMIC classification system. When utilizing the BAMIC classification system, grade 3 lesions have “high signal change of craniocaudal length of greater than 15 cm”. Whilst other criteria are used when using the BAMIC classification, the overall grade in BAMIC is assigned based on the finding that produces the highest grade. By definition as the mean length of signal change with ERSA lesions is 15.8 cm; these could be erroneously diagnosed as at least grade 3 lesions according to the BAMIC classification system [11]. This would overestimate the significance of this imaging finding and potentially lead to a delay in return to play in up to 10% of professional soccer players referred with an acute thigh injury. This could also have negative implications for management/rehabilitation.

It is noted that the BAMIC classification system includes a grade 0b, which describes, “generalized pain following unaccustomed exercise” and may show either a normal MRI appearance or patchy high signal change throughout one or more muscles [11]. The MRI appearances of grade 0b injuries have not been recorded in the literature and by definition, the professional soccer players included in this study were not partaking in unaccustomed exercise. For these reasons, grade 0b does not appear to apply to this patient cohort.

### Distinction from other clinical and imaging entities

Delayed-onset muscle soreness (DOMS) is a clinical entity characterized by tenderness, pain, and tightness of a muscle with an onset of 24 h after activity, and lasting 5–7 days [12, 13]. DOMS is typically associated with unaccustomed exercise, being less pronounced even after a single episode of activity 6 weeks prior [12]. As DOMS is a clinical entity, the MRI can be normal and there are no specific MRI features of DOMS. Supporting this, a study of Australian soccer players demonstrated poor MRI correlation to the clinical diagnosis of DOMS in the calf muscles [14]. Nonetheless, reported MRI appearances of DOMS in the literature are of feathery edema similar to a muscle strain, diffuse signal change and patchy high signal change, all of which are distinct in appearances from ERSA lesions [13–15]. The authors consider that it is improbable that the imaging findings of ERSA lesions represent a previously unrecognized imaging manifestation of DOMS as the current cohort comprises only professional soccer players.

ERSA lesions should also be distinguished from signal intensity changes in muscles on MRI immediately after muscle activity. Diffuse increases in signal intensity on T2 and STIR have been demonstrated in muscles immediately after activity, which are thought to reflect transient increases in intracellular and extracellular water content during muscle activity [16, 17]. More recently, changes in diffusion tensor imaging parameters such as fractional anisotropy have been demonstrated in muscles following muscle activity [18]. These post-activity changes in muscles are diffuse, in contrast to the findings in ERSA lesions which are of a discrete regional abnormality of the peritendinous or subfascial region.

### Limitations and future studies

The symptom profile of ERSA lesions and the impact of ERSA lesions on clinical management are difficult to assess in this retrospective cohort. The majority (67.7%) of studies exhibiting ERSA lesions in our cohort had a more significant concurrent BAMIC grade 1–4 muscle injury which is likely to account for the clinical presentation of acute thigh muscle injury. These BAMIC grade 1–4 muscle injuries were largely (81.4%) in a separate anatomic compartment to, or on the contralateral side to the ERSA lesions. Clinical management including rehabilitation in our cohort was guided by these muscle injuries rather than the ERSA lesion. Directed clinical assessment and examination of muscles with ERSA lesions, performed contemporaneous to the MRI study, could be performed in future prospective studies to characterize the symptom profile of ERSA lesions. Nonetheless, the intended purpose of this study is to provide an imaging description of ERSA as an MRI entity, recognizing that subsequent studies will be required to fully understand the clinical correlate of these lesions. ERSA lesions can occur in isolation as was seen in 10 cases in this study. It would be interesting to examine this smaller subgroup with regards to return to sport in order to better understand the significance of this MRI abnormality.

ERSA lesions are described in this study as MRI entities in soccer players with suspected acute thigh muscle injury. This specific cohort of soccer players with suspected acute thigh injuries has been used for this study as they represent the largest proportion of MRI scans in professional athletes for the study centers, allowing for a more robust analysis. The prevalence of ERSA lesions in asymptomatic athletes, in other regions of the body and on other imaging modalities such as ultrasound remains unknown. Anecdotally, the senior author has identified similar lesions in MRI studies of track and field athletes in the build-up to major competitions. It is hoped that recognition of ERSA lesions as an imaging entity will spur future studies into the clinical and pathophysiological basis of this imaging entity.

Despite increasing participation of female athletes in soccer, soccer remains a male-dominated sport at a professional level. The male bias in the cohort of patients included in this study reflects this.

## Conclusion

An intramuscular peritendinous ovoid region or a subfascial ring of faint increased signal on fluid-sensitive images was evident on MRI in 10.8% of our cohort of professional soccer players referred for suspected acute thigh muscle injury. These signal abnormalities represent a hitherto unrecognized imaging entity, for which the terminology exercise-related signal abnormality (ERSA lesion) is proposed. Although the clinical significance of ERSA lesions is not fully understood, recognition of this imaging entity is important to avoid mischaracterization of these signal abnormalities as an acute muscle injury, particularly on account of their craniocaudal length relative to the BAMIC classification system. Further work is required to assess the clinical significance of an isolated ERSA lesion when considering prognosis and return to play.
